# The Impostor Phenomenon Among Nursing Students and Nurses: A Scoping Review

**DOI:** 10.3389/fpsyg.2022.809031

**Published:** 2022-03-09

**Authors:** Ying Peng, Shao-Wen Xiao, Hui Tu, Xiao-Yun Xiong, Zhao-Jia Ma, Wen-Jun Xu, Ting Cheng

**Affiliations:** ^1^The Second Affiliated Hospital of Nanchang University, Nanchang, China; ^2^School of Nursing, Nanchang University, Nanchang, China; ^3^Peking University Shenzhen Hospital, Shenzhen, China

**Keywords:** impostor phenomenon, impostor syndrome, mental health, nursing, scoping review

## Abstract

The impostor phenomenon (IP) refers to a false internal experience of low intelligence or ability that is associated with anxiety, depression, psychological distress, and burnout. The emotions associated with the IP affect not only personal mental health but also patient care. To address this issue, we need to completely understand the prevalence of and factors related to the IP and ways to resolve/overcome IP feelings. The aim of this scoping review was to identify the existing evidence regarding the IP among nursing students and nurses and determine gaps that can be addressed in future research. We conducted our study based on the scoping review methodological framework proposed by Arksey and O’Malley (2005) and advanced by Levac et al. (2010). After searching the Embase, PubMed, Cumulative Index to Nursing and Allied Health Literature (CINAHL), Cochrane Library, Web of Science and ProQuest databases, we identified 11 studies for inclusion in this review. We found that while the IP exists in nursing students and nurses, clinical nurse specialist students and final-year nursing students are at significant risk of impostor behavior. We also found that research in the nursing field has focused on the prevalence of and factors related to the IP, but few studies have addressed ways to resolve/overcome IP feelings. Thus, research in this area should be increased. This scoping review presents research gaps that may serve as a starting point for future work on the IP in the nursing field.

## Introduction

The impostor phenomenon (IP), which is a concept that was proposed by Clance and Imes (1978), refers to “a false internal experience of low intelligence or ability.” A literature review indicates that many studies have shown that the IP exists in different populations, such as managers, resident physicians, and medical students (Rohrmann et al., 2016; Villwock et al., 2016; Gottlieb et al., 2020), but that it is also common among nursing students and nurses (Christensen et al., 2016; Gill, 2020). While the IP has been well described in other fields, research on IP in the nursing field is limited, with available studies focusing primarily on the universality of the IP (Gómez-Morales, 2021).

Studies have reported that nursing students and nurses are prone to negative psychological problems, such as stress, anxiety, and depression (Henning et al., 1998; McGregor et al., 2008; Mark and Smith, 2012; Villwock et al., 2016; Bernard et al., 2017; Al Maqbali et al., 2021). This is likely because nurses face powerful stressors on a daily basis, including conflicts with physicians, high workload, and with patients and their families (Mark and Smith, 2012; Al Maqbali et al., 2021). Negative psychological problems are prevalent among nurses, especially among nursing students (Tung et al., 2018; Wang et al., 2019). Nursing students struggle to cope with not only the stressors common in higher education institutions but also with the stressors of clinical practice. Furthermore, there is evidence that nursing students exhibit higher levels of negative psychological problems than the general student population (Bartlett et al., 2016). Although negative psychological problems are associated with many factors, many studies have demonstrated that the IP is a risk factor that impacts their mental health and that the IP is generally elevated in students compared to working professionals (Henning et al., 1998; McGregor et al., 2008; Neureiter and Traut-Mattausch, 2016; Villwock et al., 2016; Bernard et al., 2017; Brauer and Proyer, 2017, 2019). As vulnerable groups, both nursing students and nurses experience emotions associated with the IP that affect not only their personal mental health but also their level of patient care (Gómez-Morales, 2021). This may be because the IP can cause low self-esteem and even burnout (Mitchell, 2005; Villwock et al., 2016). Accordingly, it is important to pay attention to the IP among nursing students and nurses.

While there new research in this field is accumulating, there are no scoping reviews on the IP among nursing students and nurses. To understand the evidence related to the IP in the nursing field and identify the research gaps, we conducted a scoping review on the IP among nursing students and nurses.

Our review involved two research questions: (a) What evidence is there to identify, assess, and resolve/overcome the IP among nursing students and nurses? (b) What are the gaps in the evidence base? Our main concerns were the prevalence of the IP among this population, its related factors, assessment tools for identifying it, and methods and techniques for resolving or overcoming impostor feelings.

## Methods

While we applied the scoping review methodology proposed by Arksey and O’Malley (2005) and advanced by Levac et al. (2010), we did not perform the sixth step, i.e., consultation, because we studied the literature on the IP among nursing students and nurses and, thus, did not involve other stakeholders’ views on this issue.

### Identifying the Research Question

The purpose of this review was to identify the existing literature on the IP among nursing students and nurses and the research gaps within this literature. To achieve these goals, we put forward the following research questions: (a) What evidence is there to identify, assess, and resolve/overcome the IP among nursing students and nurses? (b) What are the gaps in the evidence base?

### Identifying Relevant Studies

Two researchers who have extensive evidence-based knowledge and who have each participated in systematic review searches many times searched the following databases: Embase, PubMed, CINAHL (EBSCO), Cochrane Library, Web of Science, and ProQuest. The following search terms were used: impostor syndrome/imposter syndrome/impostor phenomenon/imposter phenomenon/impostorism/imposterism and nurse*/nursing personnel/registered nurses/registered nurse*. The search time was limited to the time between establishment of the database and 17 March 2021. The language was limited to English. See Appendix 1 for the Ying Peng’s search strategy of the PubMed database. After the search was completed, we read the reference list of each article and found no new relevant articles. In addition, we discussed and identified four common core journals. Keywords in these journals were manually searched to identify missing documents, and no new documents were found.

### Study Selection

**FIGURE 1:**
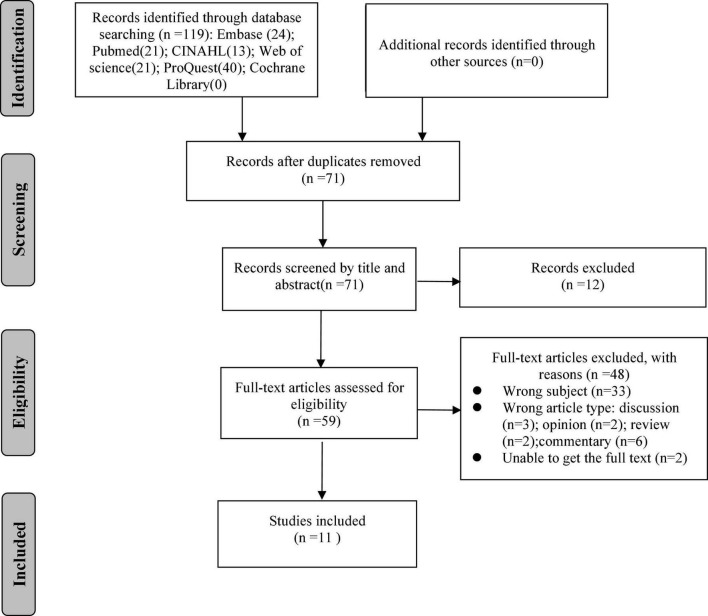
Literature screening process.

The literature inclusion criteria were as follows: (a) nursing students or nurses as the research subjects, (b) original research, and (c) any type of research design. Studies whose subjects were healthcare attendees (HCAs) or certified nursing assistants (CANs/CNAs) were excluded. Documents that did not discuss IP or opinions or debate articles were also excluded. Two researchers independently screened the articles according to the inclusion and exclusion criteria. The screening method was based on a two-stage plan (title and abstract, full text). After individual screening, the results of the two researchers were compared to determine the final included literature. If a disagreement occurred in this process, the two researchers discussed the issue together to reach consensus. If consensus could not be reached, a third person was consulted. See the literature screening flow in Figure 1.

### Charting the Data

We codesigned the data charting form based on research questions using Microsoft Excel and determined the categories of the extracted variables. Two researchers independently extracted the data from the first five documents, and then all team members met to discuss the trial of the form and determine the final categories of the extracted variables. The extracted variables included author, year, country, study type, sample size, participants, assessment tools, and major findings. The two researchers independently extracted the data and compared the two extraction results to obtain the final results. If a disagreement occurred in this process, the two researchers discussed it together to reach consensus. If a consensus could not be reached, another researcher was consulted. In the process of comparing the differences in data extraction results, the two researchers met and discussed the results three times.

### Collating, Summarizing, and Reporting the Results

We collated and summarized the research results and described them using two methods. First, the scope, nature and distribution of the included studies were summarized based on descriptive statistics. Second, we conducted a narrative review of the existing information on the aforementioned research questions. Our literature review results report is organized around these two approaches.

## Results

### Descriptive Statistics Summary

Based on the identified literature, research in this area has increased since 2016. Most studies were from the United States (Smith-Clark, 1988; Hollingsworth, 1995; Engberg, 1996; Even, 1999; Klein, 2000; Vance, 2002; Mitchell, 2005; Ares, 2018; Haney et al., 2018), and only one study was from Australia (Christensen et al., 2016). In six studies, the research subjects were nurses (Smith-Clark, 1988; Hollingsworth, 1995; Engberg, 1996; Ellerie, 1997; Even, 1999; Mitchell, 2005). In five studies, the research subjects were nursing students (Klein, 2000; Vance, 2002; Christensen et al., 2016; Ares, 2018; Haney et al., 2018). Nine were survey studies (Smith-Clark, 1988; Engberg, 1996; Ellerie, 1997; Even, 1999; Klein, 2000; Vance, 2002; Mitchell, 2005; Christensen et al., 2016; Ares, 2018), and two were mixed studies (Hollingsworth, 1995; Haney et al., 2018). See Table 1 for details.

**TABLE 1:** Basic information of the included articles.

Study	Country	Study type	Sample size, participants	Assessment tools	Major findings
Haney et al., 2018	United States	Qualitative study; survey	447 Interprofessional students	CIPS	(a) 50% of the entire group had a score of 60 or greater. (b) There is a substantial risk of IP behaviors in clinical nurse specialist students. In 14 clinical nurse specialist students, 9 scored between 41 and 60, 3 scored between 61 and 80, and 2 scored greater than 80. (c) Educational institutions with clinical nurse specialist programmes can easily incorporate content on impostor syndrome into their existing curriculum.
Ares, 2018	United States	Survey	68 Clinical nurse specialist students	CIPS	The prevalence of IP experiences at the moderate, frequent, or intense levels was 74.6%.
Christensen et al., 2016	Australia	Survey	223 Final year nursing students	CIPS	(a) Moderate IP experiences (45.1% of participants); frequent impostor feelings (33.4% of participants); intense IP experiences (>8.3%). (b) A total of 38.5% of the sample was classified as feeling like “impostors.”
Mitchell, 2005	United States	Survey	114 Nurse educators; a large random sample of a target population	CIPS	(a) This sample on the whole did not report impostor feelings (mean score < 62). (b) IP was not correlated with self-esteem (*r* = -0.013, *p* = 0.891). (c) IP was not correlated with age, gender, academic rank, number of years as a faculty member, highest degree achieved, or tenure in nurse educators (*p* = 0.553).
Vance, 2002	United States	Survey	32 Family nurse practitioner students	HIS; CIPS	(a) The mean impostor score for the total sample was a relatively low 46.6 ± 15.05. (b) IP was correlated with students’ beliefs that they would graduate with the skill level needed for advanced practice (*r* = -0.2278) and their confidence regarding their success in their future role as a nurse practitioner (*r* = -0.2972). (c) IP was correlated with numbers of years of nursing practice (*r* = -0.2904, *p* = 0.107). (d) IP was correlated with age (*r* = -0.1463, *p* = 0.424). (e) IP was correlated with familial support (*r* = -0.1915, *p* = 0.294). (f) IP was not correlated with gender or marital status (*t* = 0.54, *df* = 30) (*t* = 0.72, *df* = 28.58).
Klein, 2000	United States	Survey	181 Baccalaureate nursing students	CIPS	(a) A total of 19.33% of the students reported IP. (b) IP was not correlated with the students current age, amount of college already completed, years of previous full-time work, number of children, hours preparing for clinical, current GPA or grade on the last test. (c) IP was correlated with role function behavior and the students’ perceptions of competency (*r* = 0.427) (*p* < 0.001). (d) The study indicated that with generic senior baccalaureate nursing students, and IP has positive consequences.
Even, 1999	United States	Survey	129 RN preceptors	CIPS	(a) A total of 10% of the participants experienced moderate to intense levels of IP. (b) A significant negative correlation was found between impostorism and self-perceived qualification for the preceptor role. (c) Feelings reported by RN preceptors employed at acute care hospitals suggest that such an employment setting slightly increased the risk of feelings associated with IP.
Ellerie, 1997	United States	Survey	109 RN first assistants (RNFAs)	HIS	(a) The total RNFA sample results indicated that they did not experience IP. (b) IP was correlated with years of experience in the operating room (*r* = -0.1049) and years of experience in nursing (*r* = -0.0001).
Engberg, 1996	United States	Survey	222 Primary care providers	HIPS; CIPS	(a) The mean IP score for the total sample was a relatively low 29.2 ± 14.93. (b) IP was not correlated with personal and practice characteristics for the total group. (c) For nurse practitioners (NPs), marital status was the only characteristic found to be related to the IP score.
Hollingsworth, 1995	United States	Qualitative study; survey	527 Enterostomal therapy (ET) nurses	HIS; CIPS	(a) ET nurses had a minor intensity of IP manifestations. (b) Recent graduates of a non-traditional ETNEP showed a slightly higher incidence of IP manifestations than both experienced ET nurses and non-ET nurses. (c) As the individual respondents passed age 48 years, IP manifestations declined in intensity. (d) IP intensity was not correlated with age, gender, race/ethnicity, or programme type.
Smith-Clark, 1988	United States	Survey	110 RNs	HIS	(a) The RNs in this study demonstrated relatively low IP scale scores. (b) The possible range of scores was 0–84. The scores ranged from 4 to 58 with an overall mean of 25.50. (c) It appears that the subjects did not experience an intense level of impostor feelings. (d) This finding is similar to Topping’s results obtained with university professors (1983).

*CIPS, Clance Impostor Phenomenon Scale; HIS, Harvey Impostor Scale; IP, Impostor Phenomenon; RN, Registered Nurses.*

### Prevalence of Impostor Phenomenon in the Population

Of the 11 studies included, six studies used the Clance Impostor Phenomenon Scale (CIPS) to assess prevalence (Even, 1999; Klein, 2000; Mitchell, 2005; Christensen et al., 2016; Ares, 2018; Haney et al., 2018), three studies used both the CIPS and Harvey Impostor Scale (HIS) (Hollingsworth, 1995; Engberg, 1996; Vance, 2002), and only two studies used the HIS (Smith-Clark, 1988; Ellerie, 1997). Studies measuring the prevalence of IP among nursing students showed that it ranged from 19.33 to 100% (Klein, 2000; Vance, 2002; Christensen et al., 2016; Ares, 2018; Haney et al., 2018). Clinical nursing students and final-year nursing students were at great risk of IP (Christensen et al., 2016; Ares, 2018). Tina et al. found that the IP prevalence among 14 clinical nursing students was 100% (Ares, 2018). Martin et al. studied 223 final-year nursing students and found that the rate of IP was 86.8%, and these participants experienced moderate and higher levels of impostor feelings (Christensen et al., 2016). No study assessed the IP incidence rate of nurses.

### Factors Related to Impostor Phenomenon in the Population

The initial view was that IP mostly occurred in successful women (Clance and Imes, 1978), but most nursing literature showed that the proportion of men and women experiencing IP was similar and that gender was not statistically significantly related to IP (Smith-Clark, 1988; Hollingsworth, 1995; Engberg, 1996; Ellerie, 1997; Vance, 2002; Mitchell, 2005). There is also controversy about race as a factor of IP. Studies outside the nursing field have suggested that race has a significant impact on IP, but two nursing field studies showed that race had no correlation with IP (Smith-Clark, 1988; Hollingsworth, 1995). Several studies have found that nursing students and nurses with low self-confidence and self-esteem have increased IP incidence.

### Ways to Resolve/Overcome Impostor Phenomenon Feelings

Impostor phenomenon is considered a stable feeling (does not change over time or with situations), and IP can relate to mental health problems, such as depression, anxiety, and psychological distress (Henning et al., 1998; McGregor et al., 2008; Villwock et al., 2016; Bernard et al., 2017). Therefore, it is important to overcome impostor feelings. In our review, two studies suggested that individuals should be aware that IP is a normal experience at a certain stage and that their peers may also experience the same self-doubt (Hollingsworth, 1995; Studdard, 2002). Another study suggested that educational institutions with clinical nursing courses should incorporate IP content into their existing courses, conduct IP emotional education for students and establish mentoring relationships with students (Haney et al., 2018).

## Discussion

We identified the existing literature on IP among nursing students and nurses. Since 2016, research on IP among nursing students and nurses has seemed to increase. We found that most of the research is performed in the United States and mainly focuses on the universality of IP in this population. Notably, most of these studies are survey studies, and there are few mixed studies. Although the number of mixed studies is limited, mixed research methods can better understand the IP experience among nursing students and nurses.

Our review highlights some gaps in the existing research. First, the gold standard for IP assessment has not yet been established. Karina et al. noted that this is affected by the conceptual clarity of the IP dimensions and the lack of psychometric data (Mak et al., 2019). The assessment of IP lacks uniformity, which seriously affects the comparison between studies and thus affects the drawing of broader conclusion. Although the CIPS is the most commonly used assessment tool for researchers, the current research has not determined its advantages over other tools. This seems to be a way forward. Future confirmatory research should determine the dimensions of IP to solve the conceptual clarification of structural dimensions and report necessary psychometric data.

Second, we found that the nursing field has given little attention to the IP. Research on the IP focuses primarily on the universality of IP in the population, and thus, little is known about the impact of different activities to overcome the experience of self-doubt and its consequences, a finding that aligns with the study results of Gómez-Morales (2021).

The findings with respect to gender and race as two factors related to the IP contradict those of previous studies. In our review, none of the research results indicated that gender and race are related to the IP. A possible reason is that in the majority of studies, the respondents’ gender and race were not balanced. For example, most of the study respondents were white women. Therefore, it is recommended that future research regarding the IP in the nursing field avoid this bias by expanding the sample size to verify whether the two factors are related to the IP. In addition, the IP has a negative impact on mental health, whereas the positive impact of the IP has received little attention, which is perhaps another research direction. Future research can also link IP-related factors with their impacts on mental health to explore how a specific factor regulates the IP and mental health and to understand how they, i.e., the specific factor, the IP and mental health, interact. We also found a paucity of intervention studies, although a study outside of the nursing field noted that systematic intervention is the only method that addresses the IP (Freeman and Peisah, 2021). Based on the related evidence of the factors influencing the IP, future studies should develop customized interventions specifically for the degree of the IP and explore the effects of those interventions on impostor feelings.

Research on resolving/overcoming impostor feelings has not been fully studied. In our review, only a few studies even mentioned methods for resolving/overcoming impostor feelings (Haney et al., 2018), and those methods have not received the attention they deserve. However, as many studies have found that the IP can cause mental health problems, such as depression, anxiety, low self-esteem, and psychological distress (Henning et al., 1998; McGregor et al., 2008; Villwock et al., 2016; Bernard et al., 2017), this is a call for future research to develop methods that will resolve/overcome impostor feelings at both the individual and institutional levels and to perform confirmatory research.

Finally, it is anticipated that future studies will consider these research gaps as starting points for future studies on the IP among nursing students and nurses.

### Limitations

The process of conducting this scoping review was subject to the following limitations. First, the review was performed without the cooperation of a librarian. Second, the research subjects were directly limited to the nurses and nursing students within the searches. In addition, we reviewed literature up to March 2021, and thus, any literature published after that date was not included.

## Conclusion

It is concluded that the IP exists among nursing students and nurses. This scoping review identifies the existing literature on the IP among nursing students and nurses and identifies the gaps in the current research. We expect that follow-up studies will consider this review to be a starting point for conducting valuable research on the IP among nursing students and nurses.

## Author Contributions

YP and S-WX participated in the methodology and analysis. Z-JM, W-JX, and TC contributed to data analysis. YP, S-WX, and HT contributed to the writing. YP was the main contributor to manuscript writing. HT and X-YX took part in manuscript revision. All authors participated in designing the study, read, and agreed to the published version of the manuscript.

## Conflict of Interest

The authors declare that the research was conducted in the absence of any commercial or financial relationships that could be construed as a potential conflict of interest.

## Publisher’s Note

All claims expressed in this article are solely those of the authors and do not necessarily represent those of their affiliated organizations, or those of the publisher, the editors and the reviewers. Any product that may be evaluated in this article, or claim that may be made by its manufacturer, is not guaranteed or endorsed by the publisher.

## Appendix 1: Retrieval Strategy

**Table:**

Search	Query
#1	“imposter syndrome” [Supplementary Concept]
#2	((((”imposter syndrome”[Title/Abstract]) OR (“impostor phenomenon”[Title/Abstract])) OR (“imposter phenomenon”[Title/Abstract])) OR (“impostorism”[Title/Abstract])) OR (“imposterism”[Title/Abstract])
#3	#1 OR #2
#4	(“Nurses”[Mesh])
#5	((“Nursing Personnel”[Title/Abstract]) OR (“Registered Nurses”[Title/Abstract])) OR (“Registered Nurse*” [Title/Abstract])
#6	#4 OR #5
#7	#3 AND #6 Filters: from 1000/1/1 to 2021/3/17
